# Giant Mediastinal Mature Cystic Teratoma During Pregnancy: A Case Report

**DOI:** 10.7759/cureus.96482

**Published:** 2025-11-10

**Authors:** Daniel Jimenez-Olmedo, Ubaldo Méndez-Ríos, Jesus A Arenas-Lugo, Diego Hernández-Zamonsett, Rebeca Margarita Armenta Reyes

**Affiliations:** 1 Cardiothoracic Surgery, Instituto Mexicano del Seguro Social, México City, MEX; 2 Cardiothoracic Surgery, Instituto Nacional de Enfermedades Respiratorias Ismael Cosío Villegas, México City, MEX

**Keywords:** mature cystic teratoma, mediastinal teratoma, multidisciplinary management, pleural effusion, pregnancy, surgical resection

## Abstract

Mature mediastinal teratomas are rare, benign germ cell tumors that are typically asymptomatic. Their presentation during pregnancy is extremely uncommon, and acute manifestations with respiratory compromise are exceptional. We report the case of a 19-year-old primigravida at 16.5 weeks of gestation presenting with progressive dyspnea, severe pleuritic pain, and a massive pleural effusion. Imaging revealed a giant anterior mediastinal mass. Tumor markers, including alpha-fetoprotein (AFP) and beta-human chorionic gonadotropin (β-hCG), were elevated, raising concern for malignancy. Due to the critical mass effect on maternal respiratory function, urgent surgical resection via left thoracotomy was performed. Histopathology confirmed a mature cystic teratoma. The patient recovered well, continued her pregnancy under specialized surveillance, and delivered at 37.1 weeks of gestation without complications. This case highlights that timely surgical intervention in a multidisciplinary setting is the definitive treatment for symptomatic mediastinal teratomas in pregnancy.

## Introduction

Primary extragonadal germ cell tumors (EGGCTs) are uncommon neoplasms that arise from primordial germ cells that have failed to complete their normal embryologic migration; they account for 2-5% of all germ cell tumors. The anterior mediastinum is the most frequent extragonadal site, and mediastinal germ cell tumors have a distinct clinical spectrum compared to their counterparts. Awareness of their epidemiology and natural history is essential when evaluating mediastinal masses in young adults. Benign mature teratoma accounts for 60% of all mediastinal germ cell tumors and has an excellent prognosis after surgical excision [1-3].

Teratomas are the predominant histologic subtype among mediastinal germ cell tumors and are most often mature (benign) lesions composed of well-differentiated tissues from all three germ layers. Mature mediastinal teratomas typically demonstrate indolent growth and are frequently incidental findings; however, when they reach a large size, they can produce symptoms related to mass effect on the airways, lung parenchyma, pericardium, and great vessels. Calcifications, fat-fluid levels, and cystic components on cross-sectional imaging are commonly seen and aid the presumptive diagnosis [4-6].

Pregnancy introduces unique considerations for mediastinal teratomas. Physiologic hormonal changes and the hyperdynamic, hypervolemic state of gestation may accelerate tumor-related symptoms or unmask previously silent lesions. Additionally, interpretation of serum tumor markers is complicated during pregnancy because certain markers (for example, beta-human chorionic gonadotropin (β-hCG) and, less commonly, alpha-fetoprotein (AFP)) are affected by gestation and by concurrent maternal-fetal processes. This diagnostic ambiguity can delay definitive diagnosis or create concern for non-seminomatous malignant components, influencing clinical decision-making [7,8].

A particularly important but uncommon complication of mediastinal teratomas is spontaneous rupture into adjacent compartments. Rupture into the pleural space can produce large, sterile exudative effusions and a chemical pleuritis due to leakage of sebaceous, keratinous, or other teratomatous contents; such presentations can mimic empyema or malignant effusion and frequently prompt urgent intervention. Radiologic correlation together with pleural fluid analysis and cytology is therefore important to distinguish inflammatory/chemical effusions from infectious or metastatic causes [9,10].

Management of large symptomatic mediastinal teratomas in pregnancy requires a multidisciplinary balance between maternal life-saving treatment and fetal safety. Current evidence and expert consensus support that non-obstetric surgery, when indicated, is safest during the second trimester to minimize teratogenic risk and the likelihood of preterm labor while providing an appropriate window for adequate anesthetic and perioperative fetal monitoring. Definitive surgical resection remains the treatment of choice for mature mediastinal teratoma and is both diagnostic and curative; complete excision is associated with excellent prognosis and low recurrence when histology is benign [4,11].

The case presented herein involves a 19-year-old primigravida at 16.5 weeks of gestation who developed severe respiratory compromise from a giant anterior mediastinal mature teratoma with massive pleural effusion. This case exemplifies the diagnostic constraints imposed by pregnancy, the interpretative challenges of tumor markers in the gestational setting, and the critical role of timely multidisciplinary surgical management to optimize maternal and fetal outcomes.

## Case presentation

A 19-year-old primigravida presented with a six-month history of progressive exertional dyspnea, which had acutely worsened and was associated with severe left-sided pleuritic chest pain at approximately 15 weeks of gestation. Obstetric ultrasound at presentation confirmed fetal viability and an estimated gestational age of 15 weeks. On examination, she was alert and oriented and hemodynamically stable, with a blood pressure of 95/63 mmHg (mean arterial pressure: 73.6 mmHg), heart rate of 72 beats per minute, and respiratory rate of 16 breaths per minute. She required low-flow supplemental oxygen at 2 L per minute to maintain an oxygen saturation above 96%. Her body mass index was 20 kg/m².

An ultrasound performed bedside documented a large left pleural effusion. A central venous catheter and a left pleural drain (Pleur-evac) were placed at the sixth intercostal space and evacuated sero-hemorrhagic fluid. Unfortunately, this procedure was performed at another medical unit, and we only have the written report. Drainage produced partial symptomatic relief and improved contralateral ventilation, but subsequent radiography showed persistent atelectasis of the left hemithorax.

**Figure 1:**
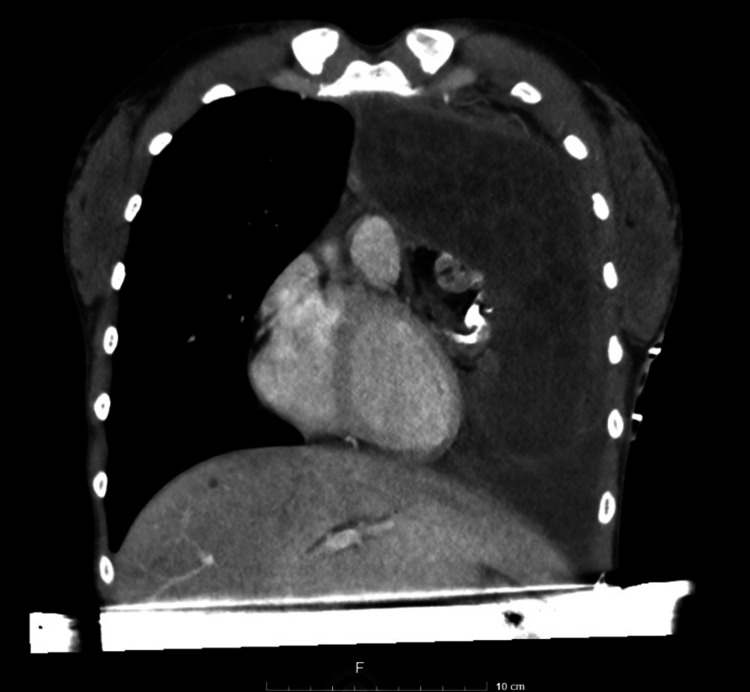
Preoperative chest computed tomography, coronal view showing a large anterior mediastinal mass expanding to the left chest.

**Figure 2:**
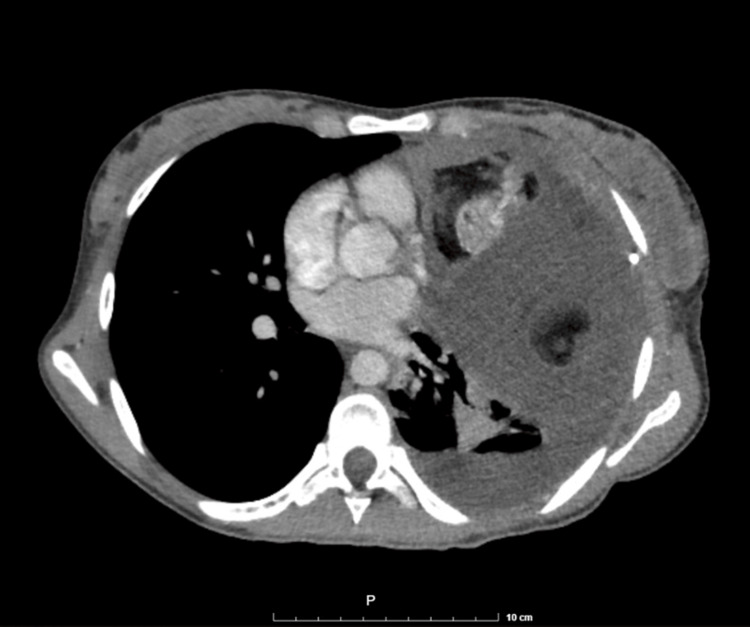
Preoperative chest computed tomography, axial view demonstrating cystic and calcified components.

Contrast chest computed tomography demonstrated a voluminous anterior mediastinal mass extending into the left hemithorax with heterogeneous composition, including cystic components and calcified foci-features highly suggestive of a teratomatous lesion. Two representative images from the patient's CT are shown (Figures 1-2).

Laboratory tests (Table 1) on admission revealed multiple, markedly elevated serum tumor markers: AFP (125.7 ng/mL; ref: <10.0), beta-hCG (10,534 mIU/mL; ref: <5.0), and CA 19-9 (1,017 U/mL; ref: <37). The beta-hCG level was significantly elevated above the non-pregnant reference range. Other serum abnormalities included low-normal hemoglobin (12.2 g/dL) and marked hyperfibrinogenemia (807 mg/dL; ref: 200-400). Analysis of the pleural fluid was consistent with a lymphocytic-predominant exudate, showing elevated lactate dehydrogenase (LDH) (637 U/L), low glucose (38 mg/dL), and approx 80% lymphocytes.

**Table 1:** Alpha-fetoprotein (AFP), beta-human chorionic gonadotropin (beta-hCG), carbohydrate antigen 19-9 (CA 19-9), and fibrinogen values were significantly elevated, although AFP and beta-hCG showed a slight decrease following pleural effusion evacuation. Pleural fluid analysis was consistent with an exudative profile (increased lactate dehydrogenase (LDH) and decreased glucose) with lymphocytic predominance.

Serum Markers	Day 1	Day 5	Reference Range
AFP (ng/mL)	125.7	99.3	<10.0
β-hCG (mIU/mL)	10,534	10,223	<5.0 (non-pregnant)
CA 19-9 (U/mL)	1,017	1,041	<37
Hemoglobin (g/dL)	12.2	11.1	12.0−15.0
Fibrinogen (mg/dL)	807	701	200−400
Pleural Fluid			
LDH (U/L)	637	-	<200
Glucose (mg/dL)	38	-	70−100
Lymphocytes (%)	≈80%	-	<20%

Following drainage (day 5), the beta-hCG and AFP levels slightly decreased (to 10,223 mIU/mL and 99.3 ng/mL, respectively), while CA 19-9 showed a modest increase to 1,041 U/mL. Throughout this perioperative course, the hemoglobin progressively declined to 11.1 g/dL.

Pleural fluid analysis showed an exudative profile (LDH increased and glucose decreased, as detailed in Table 1) with approximately lymphocytes, sterile cultures, and cytology reported as non-neoplastic (category II). This pattern was interpreted as more consistent with sterile inflammatory/chemical pleuritis from teratomatous leakage or microscopic rupture rather than bacterial empyema or pleural metastasis.

The patient presented with progressive respiratory compromise, including a near-complete collapse of the left lung, and elevated tumor markers. These findings raised concern for a possible non-seminomatous component, prompting a multidisciplinary team (cardiothoracic surgery, obstetrics, anesthesiology, and critical care) to conclude that urgent surgical intervention was indicated.

The team elected to operate during the second trimester, when fetal risk from non-obstetric surgery is relatively lower. Consequently, on April 26, 2023, at 17 weeks’ gestation, the patient underwent a left thoracotomy. For the procedure, she was intubated with a double-lumen endotracheal tube to facilitate single-lung ventilation. General anesthesia was induced and maintained using fentanyl, rocuronium, and sevoflurane.

**Figure 3:**
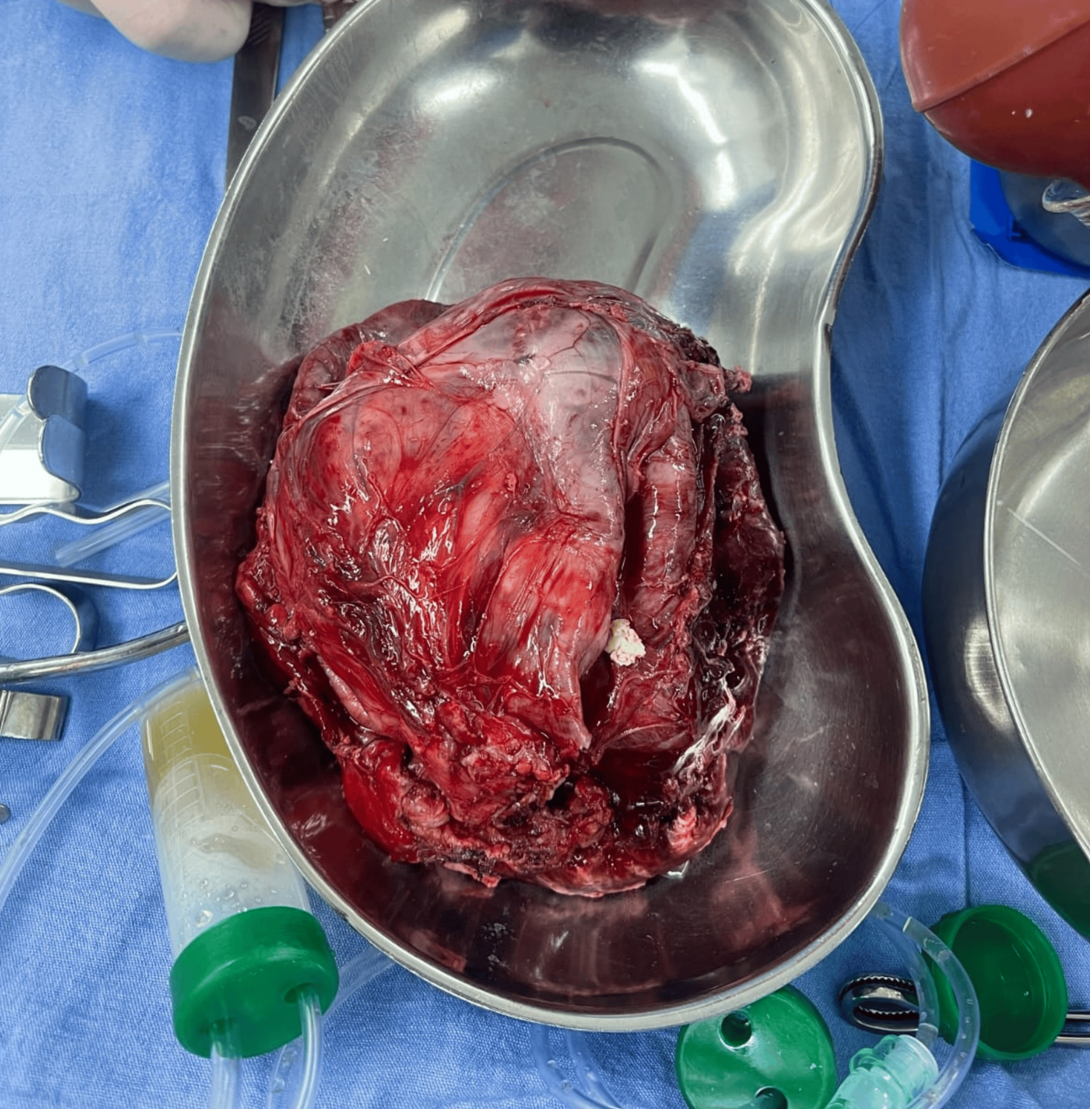
Resected intact mediastinal teratoma.

**Figure 4:**
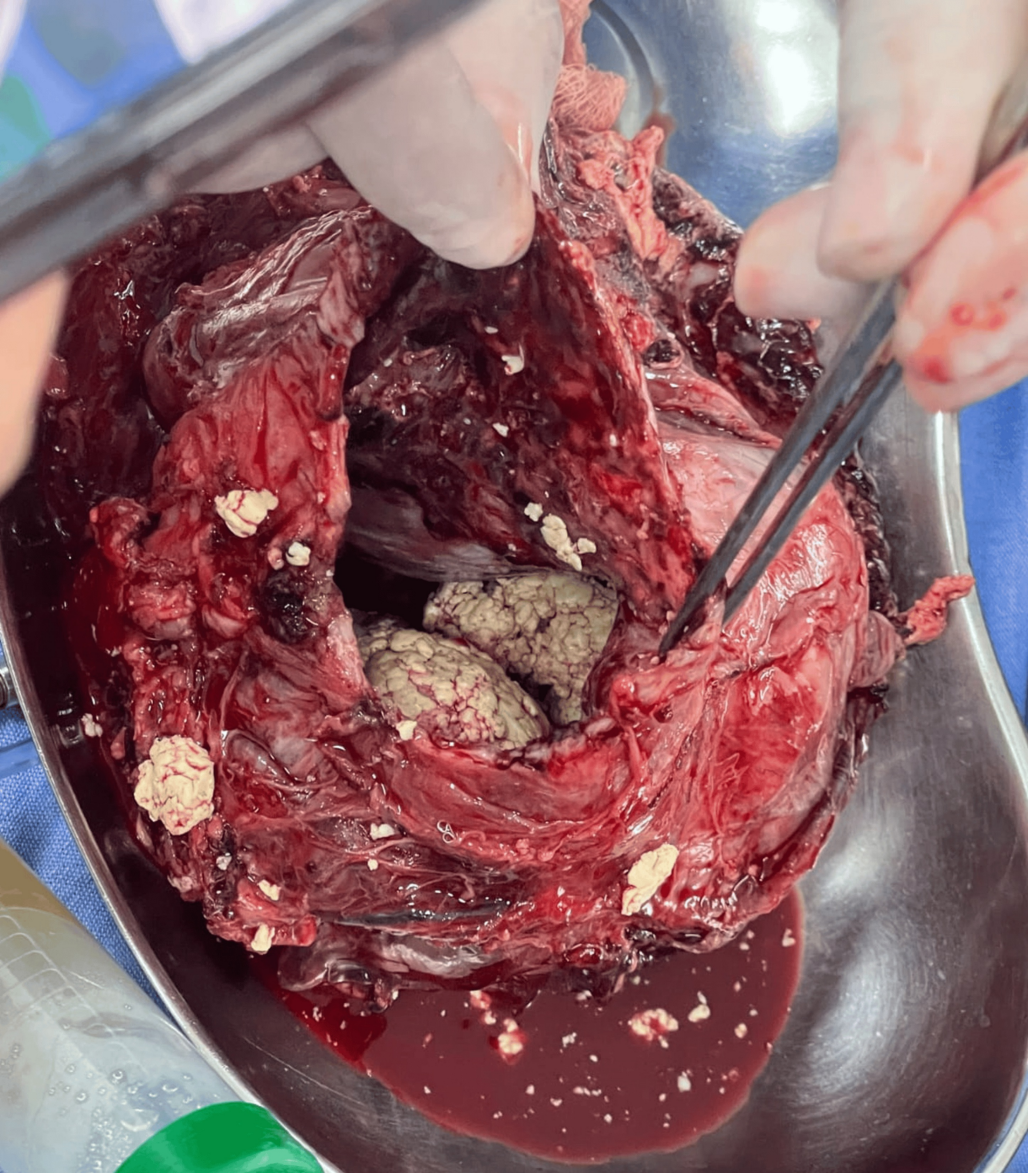
Opened tumor showing cystic contents with hair and sebaceous material.

Intraoperatively, a large left intrathoracic mass measuring approximately 16 × 12 × 10 cm was identified. The lesion was predominantly cystic, containing turbid, thick, yellowish material, with focal areas of wall thinning and discoloration suggestive of prior micro-rupture into the pleural cavity. Surrounding tissues showed localized inflammatory adhesions. The mass was meticulously dissected and removed en bloc without rupture (Figure 3). On gross inspection, the opened specimen revealed multiloculated cystic spaces filled with turbid fluid, hair, and abundant sebaceous material, features highly characteristic of a mature cystic teratoma (Figure 4).

**Figure 5:**
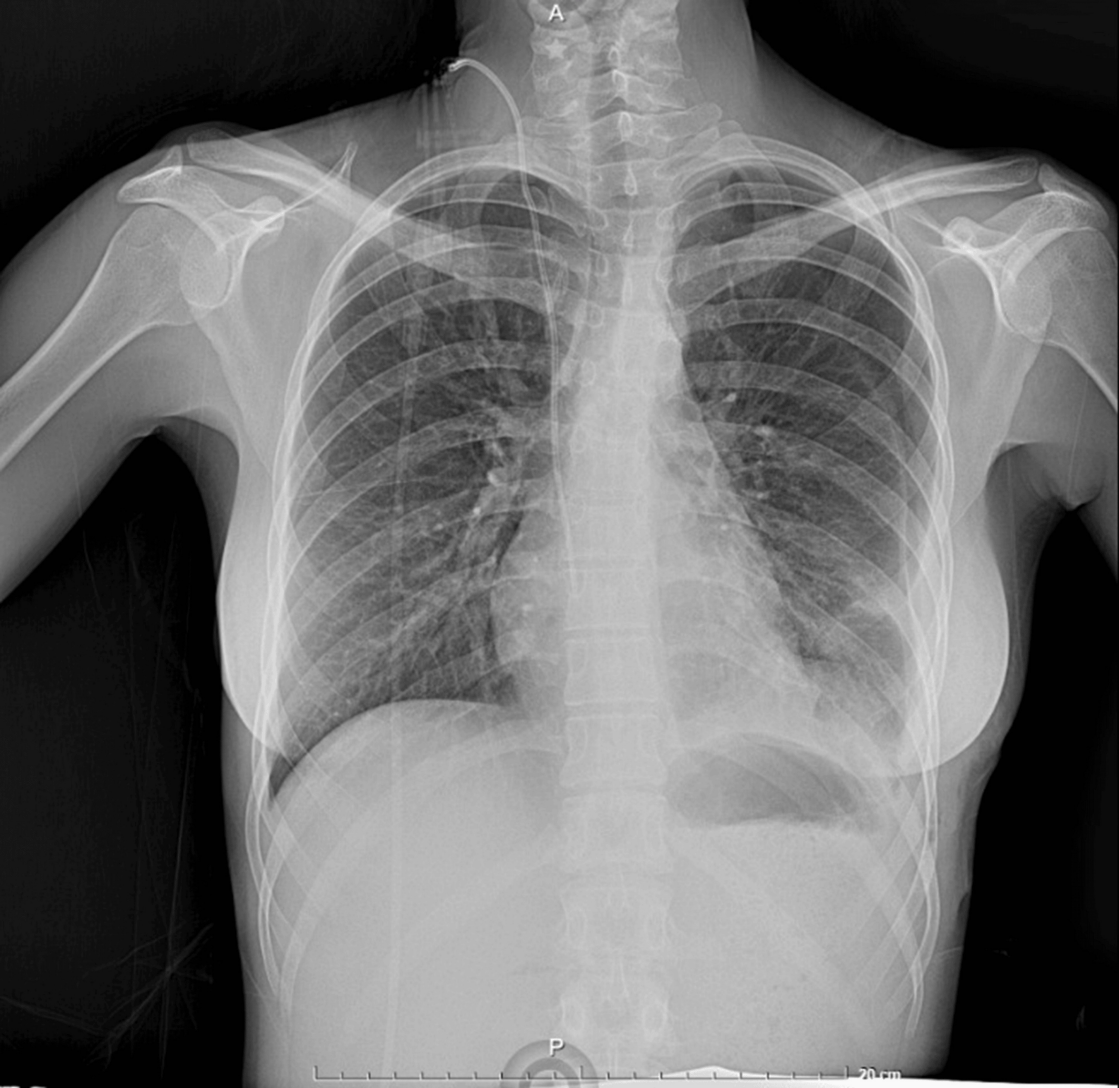
Postoperative chest radiograph showing complete lung re-expansion.

The pleural space was irrigated, and drains were placed. The immediate postoperative course in the respiratory ICU focused on optimizing maternal oxygenation and hemodynamics to preserve uteroplacental perfusion. The patient required supportive care for an unexpected postoperative hemoglobin drop but did not require reoperation and had no early surgical complications. Final histopathology confirmed a mature cystic teratoma without immature or malignant germ cell elements, obviating the need for adjuvant oncologic therapy. She was discharged home one week after surgery in stable condition and continued obstetric follow-up. The pregnancy progressed to delivery by cesarean section at 37 weeks for fetal growth restriction; both mother and neonate had satisfactory outcomes at follow-up. Postoperative chest radiograph at one week demonstrated complete re-expansion of the left lung and absence of residual mediastinal mass (Figure 5).

Throughout the clinical course, definitive surgical treatment proved pivotal: resection relieved life-threatening mass effect, provided the only reliable tissue diagnosis in the context of ambiguous tumor markers during pregnancy, and removed the source of ongoing pleural inflammation. Given the potential for rapid deterioration from airway compromise or refractory hypoxemia, the decision for prompt resection by a coordinated multidisciplinary team was central to achieving the favorable maternal and fetal outcome in this case.

## Discussion

This case reinforces the principle that definitive surgical resection is the cornerstone of management for large, symptomatic mature mediastinal teratomas and, in the setting of pregnancy, frequently the only measure that simultaneously accomplishes diagnosis and cure. The clinical course described - progressive respiratory compromise, massive sterile pleural effusion, near-complete ipsilateral lung collapse, and an AFP elevation that raised concern for a non-seminomatous element - created a time-sensitive scenario in which conservative measures alone (drainage, observation) were insufficient to secure maternal safety or to provide a definitive diagnosis. In such circumstances, operative removal is not merely optional but potentially lifesaving, because it directly reverses the mass effect, stops ongoing chemical pleural irritation, and allows histologic assessment that definitively rules out malignant components [4,6,9,10].

The pathophysiologic rationale for surgery is straightforward. Large cystic teratomas exert direct compressive forces on lung parenchyma, bronchi, and mediastinal structures; evacuation of pleural collections without removing the source may temporize symptoms but does not eliminate continued leakage of irritant teratomatous contents. Recurrent or persistent pleural contamination perpetuates inflammation, fibrothorax risk, and progressive respiratory failure. Furthermore, when tumor markers are discordant or elevated in pregnancy, the inability to exclude malignant germ cell elements noninvasively places the clinician in a dilemma: delay could allow progression of an occult malignancy, while early histologic confirmation via excision clarifies prognosis and obviates empirical adjuvant therapy if benign. Thus, resection addresses both mechanical and oncologic imperatives simultaneously [2,7,9].

Decision-making in pregnant patients must carefully balance maternal benefit and fetal risk. Several practical points underpin the clinical choice to operate at 16-17 weeks in this patient. First, the second trimester is generally regarded as the safest window for non-obstetric surgery: organogenesis is complete, teratogenic risk is lower than in the first trimester, and the chance of precipitating preterm labor is lower than in the third trimester. Second, the magnitude of maternal compromise - respiratory insufficiency with nearly collapsed lung - made expectant management unacceptably risky. Third, a multidisciplinary preoperative plan (thoracic surgeons, obstetricians, anesthesiologists, neonatologists, and critical care) optimizes intraoperative strategies (positioning, uterine protection, fetal monitoring when feasible) and postoperative care focused on maternal oxygenation and hemodynamic stability to preserve uteroplacental perfusion. Our case exemplified these principles: coordinated planning permitted definitive treatment with favorable maternal and fetal outcomes [4,11].

Surgical considerations deserve particular attention. Complete en bloc resection with careful avoidance of cyst spillage is the technical objective. Spillage of cyst contents can perpetuate pleural inflammation and increase postoperative morbidity; meticulous dissection, controlled decompression if necessary, and thorough pleural irrigation minimize this risk. When a mass is adherent to adjacent structures, experienced thoracic surgical technique and intraoperative judgment determine whether extended resection or staged procedures are required. In pregnant patients, minimizing operative time and blood loss and ensuring adequate oxygenation and normotension are critical to reduce fetal risk. Peripheral and central access planning, availability of blood products, and readiness for conversion to more extensive procedures are essential components of preoperative preparation [4,6].

Anesthesia and perioperative fetal considerations are integral to safe operative care. Anesthetic management aims to maintain maternal oxygen delivery, avoid hypotension, and minimize uterine irritability. Where feasible, intraoperative fetal heart rate monitoring can provide real-time assessment of fetal well-being, although technical limitations and gestational age sometimes preclude continuous monitoring. Postoperative care should prioritize early mobilization, aggressive pulmonary hygiene, appropriate analgesia that avoids maternal hypoventilation, and close obstetric surveillance for uterine activity or fetal concerns. In our patient, these measures helped preserve uteroplacental perfusion and contributed to an uneventful recovery and continuation of pregnancy [3,10].

Alternative or adjunctive strategies (serial drainage, percutaneous biopsy, or observation) have limited roles when mass effect is severe or marker elevation raises suspicion for malignancy. Percutaneous biopsy may be non-diagnostic in cystic lesions and carries a risk of seeding or provoking rupture. Repeated drainage can relieve symptoms transiently but does not eliminate the underlying pathology and may increase the complexity of later definitive resection. Thus, early definitive resection is preferable to prolonged temporizing measures when maternal compromise is present [9,10].

Outcomes after complete resection of mature mediastinal teratoma are generally excellent. When histology confirms a mature (benign) teratoma and margins are clear, adjuvant therapy is not indicated, and recurrence rates are low. Nonetheless, the unusual context of pregnancy and the initial marker elevation in our patient justify a cautious follow-up strategy. Follow-up should include periodic clinical assessment and interval imaging - chest radiography or computed tomography as clinically indicated - and selective tumor marker surveillance interpreted in coordination with obstetrics, given the confounding effects of gestation. Longitudinal surveillance ensures prompt detection of rare recurrence or delayed complications such as fibrothorax or persistent pleural inflammation [6].

Mediastinal mature teratomas during pregnancy are exceedingly rare, with fewer than 50 cases reported in the literature to date. Most cases are described in single case reports or small series, highlighting both the rarity of the condition and the limited high-level evidence available. Previous studies consistently demonstrate that complete surgical resection is the definitive treatment, leading to excellent maternal and fetal outcomes, whereas delayed or incomplete surgery increases the risk of complications, including pleural contamination and tumor rupture. This case also has educational value in emphasizing system-level requirements for managing complex thoracic pathology during pregnancy. Rapid access to high-dependency care, integrated multidisciplinary teams capable of balancing obstetric and thoracic priorities, and established institutional pathways for non-obstetric surgery can shorten time-to-intervention and improve outcomes. Training and preparedness are particularly important in centers that serve as referral hubs for rare conditions, such as giant mediastinal teratomas [4,11].

## Conclusions

Mediastinal mature teratoma is a rare, slow-growing tumor that often remains clinically silent until it produces compressive symptoms or complications. During pregnancy, its management requires a careful balance between maternal and fetal well-being. In this case, timely surgical resection provided definitive diagnosis and relief of cardiorespiratory compromise. Multidisciplinary coordination among thoracic surgery, obstetrics, anesthesiology, and critical care teams was essential to ensure optimal perioperative outcomes. With appropriate timing and perioperative planning, surgical management of large symptomatic mediastinal teratomas in pregnancy offers excellent maternal and fetal prognosis, with minimal risk of recurrence.
